# Overcoming MCL-1-driven adaptive resistance to targeted therapies

**DOI:** 10.1038/s41467-020-14392-z

**Published:** 2020-01-27

**Authors:** Kris C. Wood

**Affiliations:** Department of Pharmacology and Cancer Biology, Duke University, Durham, NC USA

**Keywords:** Targeted therapies, Cancer therapeutic resistance

## Abstract

Two complementary studies in *Nature Communications* define a critical role for the anti-apoptotic protein MCL-1 as a driver of adaptive survival in tumor cells treated with oncogene targeted therapies, providing a rationale for combining these agents with newly developed MCL-1 inhibitors in the clinic.

## The challenge of residual disease

Targeted cancer therapies, which inhibit the oncoproteins and underlying pathophysiological processes that drive malignant progression, have the potential to selectively eradicate tumor cells while sparing healthy tissues. This notion is supported by evidence of remarkable activity in a subset of cancer patients. However, in advanced disease settings, these agents rarely yield complete and durable responses. The ability of a subset of cancer cells within each patient to survive treatment is critical, as these cells provide the reservoir from which eventual progressive disease emerges^1^. For this reason, there is now great interest in defining upfront therapeutic strategies with the potential to reduce or eliminate residual disease. However, progress in this area has been relatively slow to date, in part because accessing and comprehensively analyzing tumors at the minimal residual disease state has been logistically challenging^1^.

Clearly, one defining feature of residual cancer cells is that unlike their drug sensitive counterparts, they fail to die following exposure to therapy. Over the past two decades, the mechanisms by which tumor cells resist death, in particular through dysregulation of the intrinsic apoptotic pathway, have been mapped in detail^2^. This tightly regulated pathway is controlled by the balance of pro- versus anti-apoptotic proteins in the mitochondria. Briefly, mitochondria contain cytochrome c, whose release into the cytoplasm causes the activation of the caspase cascades that execute irreversible, apoptotic cell death. Cytochrome c release is facilitated by the effector multidomain pro-apoptotic proteins BAK and BAX, which are activated by activator BH3-only proteins like BIM and BID to trigger mitochondrial membrane permeabilization. Given their critical importance in apoptosis regulation, it is no surprise that BAK, BAX, BIM, and BID are tightly regulated at the levels of both expression and function. For example, a key mechanism of post-translational regulation of BIM and BID is through their binding to the inhibitory BCL-2 family of multidomain anti-apoptotic proteins (e.g., BCL-2, BCL-X_L_, and MCL-1), which themselves are regulated in diverse ways by upstream signaling pathways. In recent years, researchers have become particularly interested in targeting this network because of three key advances: (1) the seemingly paradoxical observation that cancers typically maintain apoptotic competence^3^; (2) the observation that some cancer cells are particularly “primed” to undergo apoptosis relative to most normal cells in the adult^2^, and (3) the development of selective, potent, and in vivo bioavailable small molecule inhibitors of the BCL-2 family proteins (e.g., BCL-X_L_, BCL-2, and MCL-1), known as “BH3 mimetic” drugs^4,5^. In fact, one such agent—the selective BCL-2 inhibitor venetoclax—has recently shown impressive activity as a single agent in patients with hematological malignancies.

## MCL-1 as a driver of adaptive cancer cell survival

Against this backdrop, two recent studies in *Nature Communications*^6,7^ sought to define the contributions of BCL-2 family anti-apoptotic proteins to cancer cell survival in the presence of targeted therapies. In the first study, Montero and colleagues^6^ assembled a panel of 21 cancer cell lines driven by the oncogenes BRAF, KIT, EGFR, MET, ALK, or HER2. They demonstrated that siRNA-mediated suppression of MCL-1 enhanced the killing of these lines by their cognate targeted therapies more robustly than other BCL-2 family members, independent of tumor lineage, driver oncogene, or targeted therapy. This effect was confirmed using dynamic BH3 profiling and extended to clinical samples using an elegant approach combining flow cytometry-based BH3 profiling with tumor-specific antibodies to ensure that therapy-induced MCL-1 priming effects were observed in tumor cells and not just the contaminating stroma. Mechanistically, MCL-1 priming was driven by the therapy-induced loss of NOXA, a “sensitizer” BH3-only pro-apoptotic protein that acts as an endogenous inhibitor of MCL-1^2^. Specifically, NOXA levels were suppressed through destabilization of *NOXA* mRNA via binding to ZFP36, an RNA decay protein that is negatively regulated by ERK-mediated phosphorylation at serine residues 218 and 228. Inhibition of the RAF-MEK-ERK pathway in *BRAF* mutant melanoma cells led to ZFP36 S218/S222 dephosphorylation and subsequent binding to *NOXA* mRNA, triggering its decay. This event frees MCL-1 to bind and inhibit the pro-apoptotic activity of BIM, an effect that can be overcome with a selective MCL-1 inhibitor, resulting in the dramatic sensitization of cells to targeted therapy. Excitingly, this phenomenon can be exploited in vivo, as treatment of xenograft models of *BRAF* mutant melanoma with the RAF inhibitor dabrafenib induced an MCL-1 dependency in surviving tumor cells that could be exploited through subsequent treatment with the MCL-1 inhibitor S63845 to eradicate these cells, leading to tumor growth inhibition and survival that substantially exceeded what could be achieved with either agent alone^6^.

A related study by Sale and colleagues arrived at similar conclusions using complementary approaches^7^. In melanoma cell lines and tumors, they observed that the MCL-1:BCL-X_L_ ratio is considerably higher than in colorectal, lung, and pancreatic tumors. As such, MCL-1 inhibitors strongly sensitized *BRAF* and *NRAS* driven melanoma cell lines to inhibition of the RAF-MEK-ERK pathway, more so than inhibitors of BCL-2/BCL-X_L_, and more so than in ERK pathway-driven colorectal cancer cell lines. Apoptosis induction following combined RAF-MEK-ERK pathway and MCL-1 inhibition was similarly observed in primary melanoma cell lines and in xenograft tumor models, including both drug naïve and resistant patient-derived xenografts, where in all cases the combination led to more penetrant and durable responses than ERK pathway inhibition alone. Similar to the findings of Montero and colleagues, Sale and colleagues reported that cell death induced by the combination was BIM- and BAX/BAK-dependent and associated with targeted therapy-induced NOXA loss and resultant neutralization of BIM by MCL-1, an effect that could be reversed using MCL-1 inhibitors.

## Implications

Recent studies have demonstrated critical roles for BCL-X_L_ and MCL-1 as guardians of survival, particularly in solid tumors. The recent development of selective, potent, and in vivo bioavailable BCL-X_L_ and MCL-1 inhibitors, coupled with our improved understanding of the upstream pathways that regulate these proteins, provide an opportunity to exploit this observation for therapeutic benefit^4,5^. This is particularly true if the potential toxicities of these agents, like the well-known, exquisite dependence of human platelets on BCL-X_L_^4^, can be overcome using an array of creative approaches that are currently under exploration^8^. The studies by Montero et al. and Sale et al. add to a growing body of work demonstrating that oncogene targeted therapies can profoundly sensitize tumors to BCL-X_L_ and/or MCL-1 inhibition^2,9,10^. Importantly, they extend this concept, highlighting the notion that tumor lineage may serve as a template, with MCL-1 inhibitors potentially being particularly useful for the treatment of RAF-MEK-ERK pathway-driven, neural crest-derived tumors like melanoma relative to epithelial cancers arising in the lungs, colon, and pancreas. In both cellular and animal models of melanoma, both groups demonstrate that combined MCL-1 and RAF-MEK-ERK pathway inhibition yields striking therapeutic activity. Importantly, and consistent with the irreversibility of cell death, both groups report that MCL-1 inhibitors do not need to be administered chronically alongside RAF-MEK-ERK inhibitors, but rather can exert their therapeutic effects following intermittent dosing, thereby minimizing systemic toxicity. Moving forward, these studies provide a clear path for using our knowledge of lineage-encoded BCL-2 protein dependencies^3^, alongside functional assays like dynamic BH3 profiling, to select BH3 mimetic agents to administer alongside targeted therapies, then to use knowledge of the kinetics of targeted therapy-induced apoptotic priming to define intermittent dosing regimens that drive efficient tumor cell death while minimizing toxicities.

These studies also highlight the potential value of new approaches to target vulnerabilities in those tumor cells that survive upfront treatment with targeted therapies. In melanoma, the induced MCL-1 dependence described in the current studies adds to other reports describing, for example, RTK-mediated RAF-MEK-ERK reactivation^11^ and MITF-driven changes in tumor cell metabolism^12^ as mechanisms of adaptive survival, and it also complements recent studies identifying sensitivity to GPX4-mediated ferroptosis induction in cells surviving targeted therapy^13,14^. Ongoing studies to comprehensively characterize the residual disease state promise to further expand our understanding and potentially arm clinicians with therapeutic strategies to target adaptive survival mechanisms^1^. Finally, it will be interesting to understand the extent to which long-term tumor evolution can be controlled using strategies targeting adaptive survival mechanisms given that therapeutic resistance can arise not only from cancer cells employing these mechanisms, but also those with pre-existing therapeutic resistance driven by hardwired genetic mechanisms^15^.
